# Frequency-Domain Transformation of cfDNA End-Motif Profiles Enhances Robust Cancer Detection

**DOI:** 10.3390/genes17060661

**Published:** 2026-06-05

**Authors:** Xinwei Sheng, Xinming Du, Qianqian Shi, Xionghui Zhou

**Affiliations:** College of Informatics, Huazhong Agricultural University, Wuhan 430070, China; szf517@webmail.hzau.edu.cn (X.S.); dxm@webmail.hzau.edu.cn (X.D.); qqshi@mail.hzau.edu.cn (Q.S.)

**Keywords:** cell-free DNA, cancer detection, discrete Fourier transform, end-motif, liquid biopsy

## Abstract

**Background/Objectives**: Cell-free DNA (cfDNA) end-motifs (EDMs) are promising fragmentomic features for noninvasive cancer detection; however, their diagnostic utility may be limited by background signals from abundant hematopoietic-derived cfDNA fragments. Existing EDM-based approaches, including the Motif Diversity Score (MDS) and classifiers based on raw motif frequencies, often show limited robustness across different datasets. **Methods**: To address this limitation, we developed a frequency-domain analytical framework based on the Discrete Fourier Transform (DFT), converting k-mer EDM frequency profiles into amplitude spectral features. We further constructed a stacking-based Ensemble Spectral Model (ESM) integrating multi-scale spectral features from 4–6-mer EDMs. **Results**: The framework was evaluated using 1782 plasma cfDNA samples from four independent studies comprising six datasets. Raw EDM profiles showed extremely high similarity between cancer and non-cancer samples (mean Spearman *R* = 0.999). Following DFT transformation, amplitude spectra showed improved separability between groups. Across datasets, the ESM achieved a mean AUC of 0.843, representing a 15.0% improvement over raw 4-mer EDM-based SVM models and a 56.4% improvement over the MDS. At 95% specificity, mean sensitivity reached 0.585, exceeding those of the raw EDM (0.418) and MDS (0.195). Frequency-guided motif attribution further linked spectral features to sequence-level motif patterns and potential regulatory programs. **Conclusions**: Frequency-domain transformation improves the representation of cfDNA EDM profiles and provides a robust analytical framework for cross-dataset cancer detection.

## 1. Introduction

The escalating global cancer burden necessitates the development of efficient, cost-effective, and minimally invasive diagnostic tools to improve patient survival and prognosis [1,2]. Liquid biopsy has emerged as a transformative modality, offering non-invasive advantages that traditional tissue biopsies cannot match [3,4]. Among various circulating biomarkers, cell-free DNA (cfDNA)—comprising DNA fragments released into the bloodstream via apoptosis, necrosis, or active secretion—has garnered significant attention [5,6,7]. cfDNA retains critical genetic and epigenetic information from its tissue of origin, including somatic mutations, copy number variations, and methylation patterns, enabling the capture of systemic physiological states through simple blood tests [8,9,10].

Advances in high-throughput sequencing have catalyzed the field of cfDNA fragmentomics [11,12]. Due to the protection afforded by nucleosomal structures, cfDNA exhibits non-random fragmentation patterns that reflect the systemic pathological state of source cells [13,14]. A prominent feature in fragmentomics is the cfDNA end-motif (EDM), defined as the specific nucleotide sequence preferences (typically 4-mers) at fragment termini [15]. EDM formation is governed by the interplay of intracellular chromatin structure, DNA methylation, and the activities of specific nucleases such as DNASE1L3 and DNASE1 [16,17,18]. This biological grounding underpins the significant clinical utility of EDM in non-invasive cancer detection. For instance, in hepatocellular carcinoma (HCC), metrics such as the Motif Diversity Score (MDS) enable robust discrimination from healthy controls [15]. Systematic benchmarks have demonstrated that the EDM often outperforms other fragmentomic features [19,20,21], with advanced ensemble models achieving superior accuracy across diverse cancers [22,23,24].

Despite this potential, existing EDM analysis methods face formidable challenges in clinical translation due to limited generalizability across diverse datasets. Our research reveals that both MDS and supervised EDM classifiers exhibit high performance instability; even extending the widely used 4-mer features to 6-mers provides marginal diagnostic gains. Furthermore, previous evaluations indicate that raw EDM features often perform poorly in external validation, sometimes underperforming other fragmentomic markers [19,21]. We hypothesize that these issues may be related to “signal submergence”, whereby abundant hematopoietic-derived background signals in cfDNA may obscure subtle tumor-associated variations within EDM profiles [9,25,26], limiting the ability of raw EDM frequencies to directly reflect pathological differences. Additionally, most existing methods rely heavily on prior biological knowledge and intensive parameter tuning [19,21,27], which limits the development of universal and efficient pan-cancer diagnostic tools.

**Figure 1:**
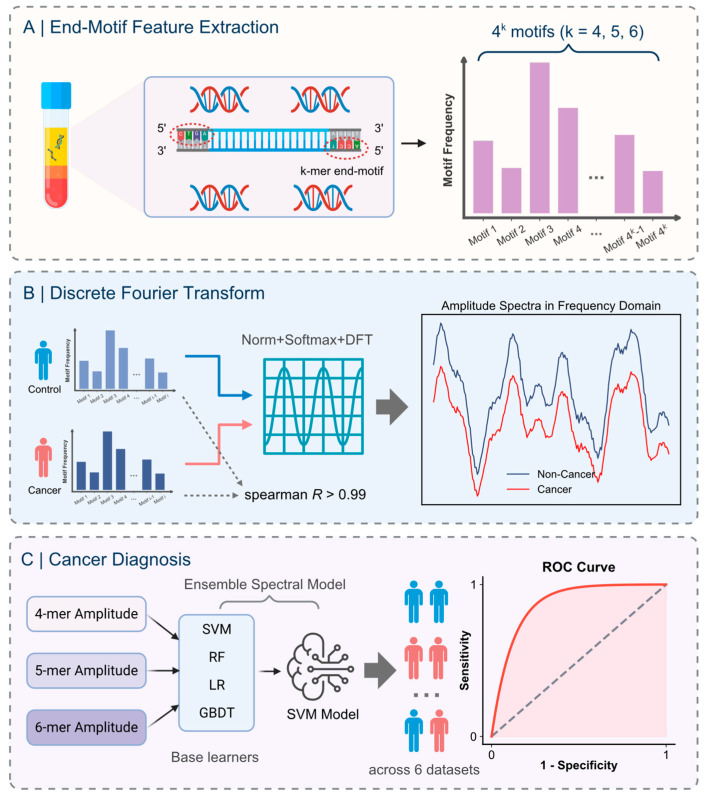
Frequency-domain analytical framework for cfDNA end-motif (EDM) analysis: (**A**) Extraction of k-mer EDM features from the 5′ termini of cfDNA fragments, generating sequence-domain representations (k = 4, 5, 6). (**B**) Transformation into the frequency domain using Discrete Fourier Transform (DFT). After normalization and nonlinear scaling, DFT is applied to the EDM profiles to generate amplitude spectra. (**C**) Construction of the Ensemble Spectral Model (ESM) for cancer diagnosis. Base learners, including support vector machine (SVM), random forest (RF), logistic regression (LR), and gradient boosting decision trees (GBDT), are trained on multi-scale spectral features. Their predicted probabilities are concatenated and used as input to a secondary SVM classifier in a stacking-based framework.

To overcome these limitations, we introduce a frequency-domain analytical framework based on the Discrete Fourier Transform (DFT), which treats k-mer end-motif distributions as discrete one-dimensional signals (Figure 1). By projecting these profiles into the frequency domain, amplitude spectra enhance the separation between cancer and non-cancer samples. Validated on 1782 samples across four independent studies comprising six datasets, our results show that spectral-based features significantly enhance diagnostic accuracy and cross-dataset stability over traditional raw-feature models, establishing a robust computational foundation for reliable liquid biopsy.

## 2. Materials and Methods

### 2.1. Study Datasets and Data Preprocessing

To evaluate the robustness of EDM features across datasets, we analyzed 1782 plasma cfDNA samples (1126 controls and 656 cancer) integrated from four independent whole-genome sequencing (WGS) studies. These datasets included: (i) the Mathios et al. lung cancer study, comprising the LUCAS dataset (*n* = 287) and an independent validation dataset (*n* = 431) (EGA accession: EGAS00001005340) [28]; (ii) the Yu et al. gastric cancer study, comprising the study dataset (*n* = 249) and the validation dataset (*n* = 167) (NGDC accession: PRJCA020703) [29]; and (iii) the Cristiano et al. pan-cancer (*n* = 423) [30] and (iv) Jiang et al. hepatocellular carcinoma (*n* = 225) datasets [31], both retrieved from FinaleDB [32]. Details for all datasets are provided in Table 1.

For model evaluation, the Mathios LUCAS and Yu study datasets were used for repeated cross-validation, while the corresponding Mathios independent and Yu validation datasets were used for external validation. The Cristiano and Jiang datasets were evaluated by repeated cross-validation only, as no matched independent validation datasets were available.

To ensure analytical consistency across heterogeneous sources, all cfDNA fragmentomic analyses were strictly performed based on the GRCh37 (hg19) reference genome. For the Cristiano and Jiang datasets, fragment genomic coordinates and mapping qualities were obtained in BED format from FinaleDB [32]. Conversely, sequencing data for the Mathios and Yu datasets were retrieved in BAM format from EGA and NGDC. Using the PySam (v0.23.0) package, we implemented a standardized preprocessing pipeline for BAM files to: (i) exclude PCR duplicates, unmapped reads, and sequences failing quality control and (ii) remove secondary and supplementary alignments to mitigate multi-mapping bias. Subsequently, all cfDNA fragments across all datasets were subjected to a final dual-filtering process, retaining only those with a length between 20 and 600 bp and a mapping quality (MAPQ) score of ≥30.

### 2.2. End-Motif (EDM) Feature Extraction and Motif Diversity Score (MDS) Calculation

Following preprocessing, EDM features were extracted by calculating the frequencies of all possible k-mer sequences (*k* = 4, 5, 6) at the 5′ termini of cfDNA fragments. For each sample, the frequency of motif *i* ($P_{i}$) was determined to construct a feature vector of dimension 4*^k^* (i.e., 256, 1024, or 4096). We subsequently computed the MDS using the normalized Shannon entropy [15]:

(1) $$MDS=-\sum_{i=1}^{N}P\left(i\right)\cdot {log}_{N}\left(P\left(i\right)\right),$$

where *N* = 4*^k^*.

### 2.3. Signal Transformation and Spectral Feature Extraction

To enhance subtle differences in EDM profiles, k-mer frequency distributions were treated as discrete one-dimensional signals, with the motif dictionary order (e.g., AAAA to TTTT for 4-mers) defining the signal axis. Prior to transformation, each motif frequency vector was standardized using Z-score normalization to improve comparability across samples. A softmax transformation was then applied across motif dimensions within each sample to obtain a normalized motif-weight representation. The resulting signals were then transformed into the frequency domain using the numpy.fft.fft function from the NumPy library (v1.26.4). The Discrete Fourier Transform (DFT) is defined as:

(2) $$X\left(k\right)=\sum_{n=0}^{N-1}x\left(n\right)\cdot e^{-j\frac{2\pi }{N}nk},\;k=0,1,\ldots ,N-1,$$

where *x*(*n*) represents the preprocessed motif signal, *X*(*k*) denotes the complex spectral coefficient and *N* denotes the motif dimensionality (*N* = 4*^k^*). We extracted the amplitude spectrum |*X*(*k*)| as the primary features for downstream analysis, as it reflects the energy distribution of motif patterns across frequencies. Phase information was computed using the NumPy angle function for subsequent reconstruction and interpretability analyses. Due to the conjugate symmetry of real-valued inputs, only the first *N*/2 frequency components (within the Nyquist frequency) were retained. Furthermore, the zero-frequency (DC) component was discarded, as its normalized constant value lacks discriminatory power for classification.

For comparative evaluation, Discrete Cosine Transform (DCT) and wavelet transformation were additionally applied to the raw EDM frequency profiles, followed by the same downstream modeling procedure used for DFT-derived features. Detailed implementation parameters for the comparative transformation methods are provided in the Supplementary Methods S1.

### 2.4. Diagnostic Model Construction and Evaluation

To establish a robust diagnostic framework, we evaluated multiple baseline machine learning models using amplitude spectral features, including Support Vector Machine (SVM), Random Forest (RF), Logistic Regression (LR), and Gradient Boosting Decision Trees (GBDT), implemented using the scikit-learn library (v1.7.1) [33]. Unless otherwise specified, all models were trained using default parameters. Detailed model implementation and hyperparameter settings are provided in Supplementary Methods S2.1.

Model performance was evaluated using repeated stratified 10-fold cross-validation (10 repetitions) implemented with RepeatedStratifiedKFold from scikit-learn, with a fixed random seed of 42 (Figure S1). For each dataset, samples were divided into ten stratified folds while preserving the proportion of cancer and non-cancer samples. In each repetition, nine folds were used for training, and the remaining fold was used for validation. Out-of-fold predictions from the ten validation folds were aggregated to generate the result for one repetition, and the average performance across ten repetitions was reported as the final cross-validation performance. This procedure generated 100 trained models for each analysis. For independent validation, the 100 models generated from repeated cross-validation were applied to the corresponding external validation dataset. Predicted probabilities from all models were averaged for each sample and used as the final model output. Model fitting was performed independently within each training split to avoid information leakage.

To improve robustness and integrate multi-scale information, we developed an Ensemble Spectral Model (ESM) using a stacking-based architecture (Figure S2). For each combination of k-mer feature (4-, 5-, and 6-mer) and classifier (SVM, RF, LR, and GBDT), model training followed the repeated stratified 10-fold cross-validation procedure described above, resulting in 100 models per combination. For each sample, out-of-fold predicted probabilities from the 10 repetitions were aggregated by averaging to generate a single score. Thus, each sample obtained 12 scores in total, corresponding to 3 feature sets × 4 classifiers. These scores were concatenated into a meta-feature vector and used to train a secondary SVM meta-classifier under the same repeated cross-validation framework. For independent validation, base-model scores were generated using the corresponding trained models, and predictions from the secondary SVM models were averaged to produce the final ESM score.

To assess the effect of motif ordering on DFT-derived features, permutation-based validation was performed by randomly shuffling motif indices 1000 times prior to DFT transformation. SVM models were then constructed using the transformed features, and AUC values from all permutations were collected for evaluation. Detailed implementation of the permutation procedure is provided in Supplementary Methods S2.2.

Model performance was primarily evaluated using the area under the receiver operating characteristic curve (AUC). For EDM-based SVM models, amplitude-based SVM models, and the ESM, sensitivity at a fixed specificity of 95% was additionally calculated. Both AUC and sensitivity were reported with 95% confidence intervals (95% CI). Model calibration and clinical utility of the ESM were additionally evaluated using calibration curves and decision curve analysis. Detailed procedures for confidence interval estimation are described in Supplementary Methods S2.3.

### 2.5. Tumor Fraction Estimation

The tumor fraction of each cfDNA sample was estimated using ichorCNA (v0.2.04) [34] by employing the same parameters as in the previous study [19]. Tumor fraction data were not available for the Yu study and Yu validation datasets. For downstream analyses, samples were stratified into low (≤2%), medium (>2% to ≤15%), and high (>15%) tumor fraction groups.

### 2.6. Frequency-Guided Motif Attribution and Functional Annotation

Frequency-guided motif attribution analysis was performed to associate frequency-domain features with sequence-level motif patterns. Using the Mathios LUCAS dataset, statistical comparisons between cancer and non-cancer samples were conducted for each frequency component of the 4-mer amplitude spectra using the Wilcoxon rank-sum test. *p* values were adjusted for multiple testing using the Benjamini–Hochberg (BH) method. Frequency components with false discovery rate (FDR) < 0.05 were retained, whereas non-significant components were set to zero before inverse transformation.

Filtered frequency-domain signals were reconstructed into motif space using the numpy.fft.ifft function. Motif-level differential analysis between cancer and non-cancer samples was then performed using the Wilcoxon rank-sum test, followed by BH correction. Differential motifs with FDR < 0.001 were retained for downstream analysis.

The identified motifs were mapped to candidate transcription factors (TFs) using motif similarity search against the HOCOMOCO v14 database [35] via the Tomtom tool (MEME Suite v5.5.8) [36]. Tomtom was run with default parameters, and motif matches with q-value < 0.5 were retained. TF-regulated target gene networks were retrieved from the TRRUST v2 database [37], and Gene Ontology (GO) enrichment analysis was performed using the clusterProfiler package (v4.10.0) [38] in R (v4.3); significant GO terms were identified using BH-adjusted *p* values (FDR < 0.05).

### 2.7. Statistical Analysis

Statistical comparisons between two independent groups were performed using the Wilcoxon rank-sum test. Comparisons among three or more groups were conducted using the Kruskal–Wallis test. Associations between variables were assessed via the Spearman rank correlation coefficient. Differences between receiver operating characteristic (ROC) curves were evaluated using the DeLong test. Unless otherwise specified, all statistical tests were two-sided, and significance was established at *p* < 0.05. Computational analyses were implemented in Python (v3.10) utilizing the SciPy library (v1.14.1) [39].

## 3. Results

### 3.1. Cross-Dataset Generalization Limitations of Raw EDM Features

**Figure 2:**
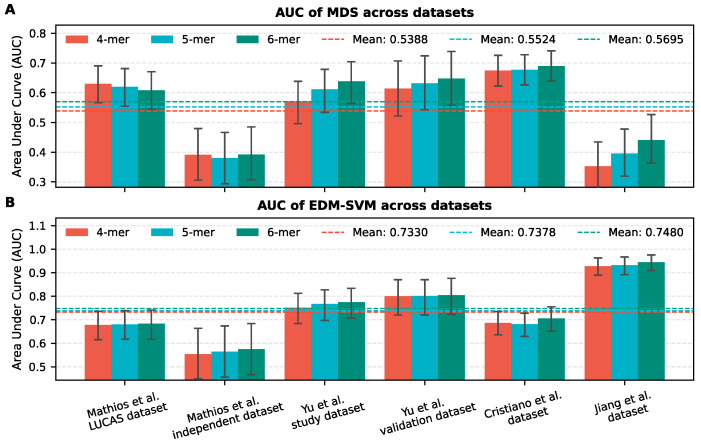
Diagnostic performance of multi-scale raw EDM features across datasets. (**A**) Area under the receiver operating characteristic curve (AUC) of the motif diversity score (MDS). (**B**) AUC of SVM classifiers based on raw EDM frequency features. Error bars above each bar indicate 95% confidence intervals (95% CI).

The Motif Diversity Score (MDS), a global entropy-based metric used to characterize the stochasticity of cfDNA fragmentation, exhibited significant instability across the six evaluated datasets (Figure 2A and Figure S3 and Table S1). For 4-mer motifs, MDS performance was particularly inconsistent; while it achieved an AUC of 0.674 in the Cristiano dataset, it dropped to 0.353 and 0.391 in the Jiang and Mathios independent datasets, respectively. This “direction-flipping” phenomenon—where metric trends reverse relative to cancer status—highlights the instability of model-free global metrics across datasets and suggests potential influences from dataset-specific biological or technical variation. Furthermore, expanding the motif length from 4 to 6-mers yielded negligible diagnostic gains, with mean AUCs of 0.539, 0.552, and 0.570, respectively, despite an exponential increase in feature dimensionality.

Supervised learning via SVMs trained on raw EDM frequencies improved diagnostic performance but failed to overcome the underlying signal submergence (Figure 2B and Figure S4 and Table S2). Compared with the MDS, raw EDM-based SVM models showed only limited performance gains overall (Table S3). Notably, in the Cristiano dataset, DeLong tests revealed no significant differences between MDS and SVM models across 4–6-mer features, suggesting that classifier construction based on raw EDM frequencies does not necessarily yield statistically significant diagnostic improvement. Although some models reached high accuracy (e.g., 4-mer AUC of 0.928 in the Jiang dataset), performance remained below the 0.7 threshold in three of the six datasets. A critical limitation is the prominent “generalization gap” observed during independent validation. For instance, in the Mathios lung cancer study, the cross-validation AUC of 0.678 in the LUCAS dataset dropped to 0.554 in the independent validation dataset. This degradation indicates that classifiers trained on raw frequencies are prone to over-fitting to dataset-specific noise rather than capturing intrinsic pathological signals. Additionally, increasing motif length provided marginal utility, with the global average AUC rising only slightly from 0.733 (4-mer) to 0.738 (5-mer) and 0.748 (6-mer).

Collectively, these findings suggest that current EDM-based approaches have limited robustness across datasets, motivating the exploration of alternative computational strategies for EDM feature representation and cancer detection.

### 3.2. Frequency-Domain Transformation Enhances the Separability of EDM Profiles

To illustrate the preprocessing prior to frequency-domain analysis, we visualized the EDM feature processing workflow (Figure S5). Z-score normalization preserved the overall distribution pattern while rescaling feature values, whereas softmax transformation enhanced high-contribution motifs and suppressed low-contribution motifs, producing a denoising-like effect before DFT.

**Figure 3:**
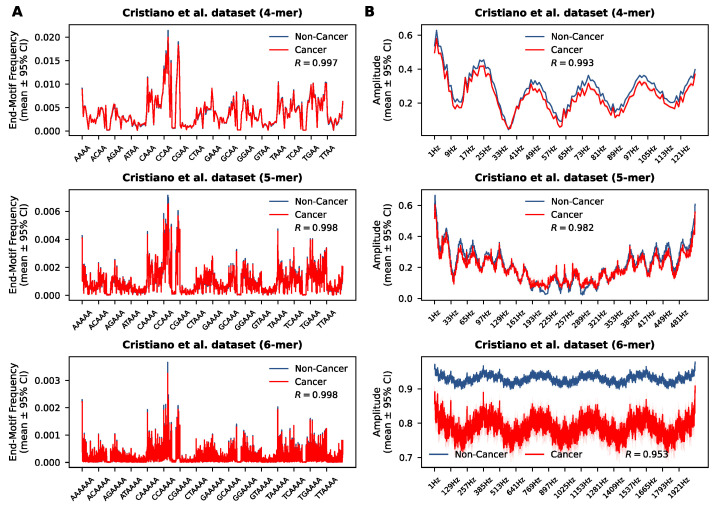
Frequency-domain transformation of EDM profiles in the Cristiano dataset. (**A**) Mean raw EDM frequency profiles of cancer and non-cancer samples across 4-mer, 5-mer, and 6-mer motifs. (**B**) Corresponding amplitude spectra after DFT. *R* indicates the Spearman correlation coefficient between the mean feature profiles of cancer and non-cancer samples. All mean feature lines shown in the figures are accompanied by shaded areas representing 95% CI.

Using the Cristiano dataset as a representative example, we applied DFT to 4-, 5-, and 6-mer EDM features. In the sequence domain (Figure 3A), raw EDM frequency profiles of cancer and non-cancer individuals showed extremely high similarity and substantial overlap, with an average Spearman correlation coefficient of 0.998 across 4–6-mer motifs. This indicates that EDM profiles in cancer patients remain highly similar to those in non-cancer individuals. Given that plasma cfDNA in non-cancer individuals is predominantly derived from hematopoietic cells [26], trace tumor-derived signals are likely masked by dominant physiological background signals, resulting in a “signal submergence” effect that limits direct extraction of pathological information from raw frequency data.

After DFT transformation, the resulting amplitude spectra showed clearer separation between cancer and non-cancer samples (Figure 3B). In the Cristiano dataset, the Spearman correlation coefficients decreased to 0.993, 0.982, and 0.953 for 4-, 5-, and 6-mer motifs, respectively. Similar patterns were also observed across other datasets (Figures S6–S10), indicating that frequency-domain representation enhances differences between cancer and non-cancer EDM profiles.

Notably, spectral trends showed dataset-specific heterogeneity. For 4-mer motifs, amplitudes in non-cancer individuals were generally higher than those in cancer patients across the Mathios, Yu, and Cristiano datasets, whereas the Jiang dataset showed the opposite pattern. This heterogeneity may partially explain the MDS “direction-flipping” phenomenon observed in the Jiang dataset. Furthermore, the separation of amplitude spectra became increasingly pronounced with increasing motif length, with most datasets showing a consistent “non-cancer-higher-than-cancer” trend. Together, these findings suggest that frequency-domain transformation improves the separability of EDM profiles and provides a more informative representation for downstream cancer detection.

### 3.3. Evaluation of Signal Transformation Methods and Modeling Strategies

**Figure 4:**
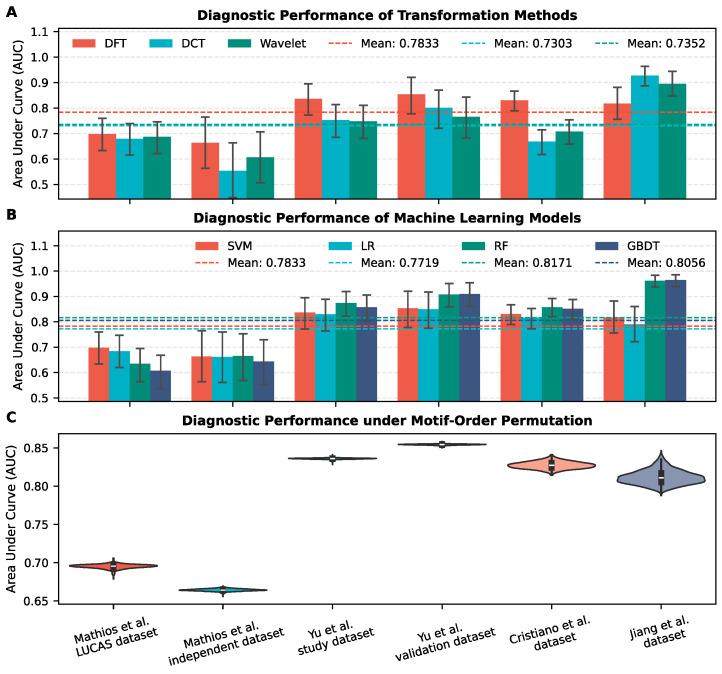
Evaluation of signal transformation methods and modeling strategies based on 4-mer EDM features. (**A**) Comparison of diagnostic performance among features generated by DFT, Discrete Cosine Transform (DCT), and wavelet transform using SVM. (**B**) Comparison of diagnostic performance among different machine learning models trained on 4-mer amplitude spectrum features, including SVM, LR, RF, and GBDT. (**C**) Distribution of AUC values from 1000 motif-order permutations of 4-mer EDM features followed by DFT transformation and SVM classification.

To evaluate the rationale for selecting DFT, we compared it with DCT and wavelet transform using 4-mer EDM features, followed by SVM classification under the same evaluation procedure. As shown in Figure 4A, DFT outperformed the other two methods in five of the six datasets, with Jiang being the only exception. Across all datasets, DFT achieved the highest mean AUC (0.783), exceeding those of DCT (0.730) and wavelet transform (0.735). Although DCT and wavelet transform performed well in the Jiang dataset, both showed relatively poor performance in the validation datasets from the Mathios and Yu studies. Similar trends were also observed for 5-mer and 6-mer features, where DFT consistently achieved the best average performance (Figure S11 and Table S4).

We next assessed whether model selection influenced diagnostic performance in the spectral domain. Multiple classifiers were evaluated using 4-mer amplitude spectral features, including SVM, LR, RF, and GBDT. As shown in Figure 4B, LR showed the lowest overall performance, with a mean AUC of 0.772. RF and GBDT achieved higher mean AUCs of 0.817 and 0.806, respectively, but exhibited substantial variability across datasets, with relatively poor performance in the Mathios LUCAS dataset and markedly higher values in the Jiang dataset. In comparison, SVM achieved a slightly lower mean AUC (0.783) but demonstrated more consistent performance across datasets. Similar patterns were observed for 5-mer and 6-mer amplitude spectral features (Figure S12 and Table S5). Notably, SVM achieved the highest mean AUC for 6-mer features, suggesting better adaptability to higher-dimensional spectral representations. Given that cross-dataset robustness is a primary objective of this study, SVM was selected as the representative baseline model.

Finally, we evaluated whether DFT performance was affected by motif ordering. By randomly shuffling motif order prior to DFT and reconstructing SVM models, we observed narrow and stable AUC distributions across all datasets (Figure 4C and Table S6). Moreover, the AUC obtained using the default lexicographic motif order was comparable to the average performance observed across permutations (Table S7), supporting the use of the default motif ordering in subsequent analyses.

### 3.4. Enhanced Cancer Detection via the Amplitude Spectra

**Figure 5:**
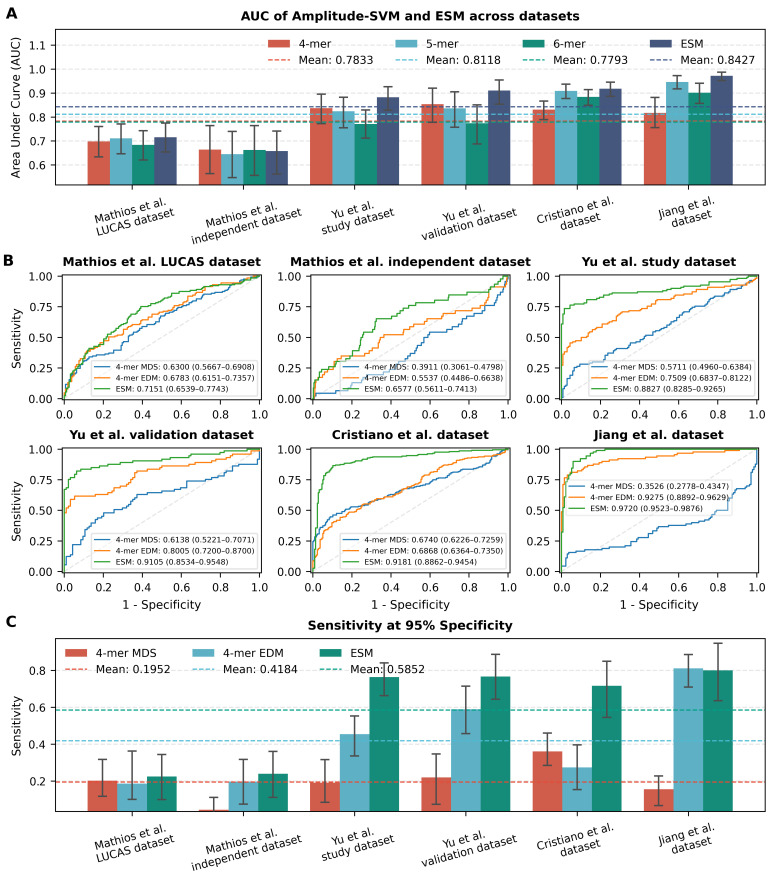
Performance comparison of the ESM and baseline methods across datasets. (**A**) Comparison of diagnostic performance using amplitude spectral features. (**B**) Receiver operating characteristic (ROC) curves comparing ESM with baseline methods, including 4-mer MDS and SVM models based on raw 4-mer EDM features. (**C**) Sensitivity at 95% specificity for ESM and baseline methods.

Given the distinct spectral separation observed in the frequency domain, we further evaluated the diagnostic performance of DFT-derived amplitude features across different k-mer lengths (Figure S13 and Table S8). In most datasets, SVM models based on amplitude spectra outperformed those based on raw EDM frequencies (Figure S14 and Table S9), while the remaining comparisons showed no significant differences by the DeLong test except for the 4-mer feature in the Jiang dataset. The average AUCs for 4-, 5-, and 6-mer amplitude spectra were 0.783, 0.812, and 0.779, respectively (Figure 5A), corresponding to improvements of 6.9%, 10.0%, and 4.2% over raw-frequency models. Although 5-mer achieved the highest overall performance, the optimal k-mer varied across datasets, suggesting complementary diagnostic information across motif scales.

To integrate these multi-scale advantages and reduce feature-specific variability, we developed the ESM using a stacking strategy. Base learners, including SVM, RF, LR, and GBDT, were trained on 4-, 5-, and 6-mer amplitude features, and their predicted probabilities were used as input to a secondary SVM classifier. The ESM achieved a global average AUC of 0.843, outperforming all single-scale amplitude-based models and showing stable diagnostic performance across datasets (Figure 5A and Table S10). In the Mathios independent and Yu validation datasets, where raw EDM models previously exhibited substantial generalization gaps, the ESM achieved AUCs of 0.658 and 0.911, representing improvements of 18.8% and 13.7%, respectively, over the raw 4-mer EDM model (Figure 5B).

Overall, compared with the 4-mer raw EDM model, the ESM showed significant improvement in five of the six datasets, with the Mathios LUCAS dataset being the only exception (DeLong test, *p* < 0.05; Figure S15 and Table S11). The ESM improved the average AUC by 15.0% compared with 4-mer raw EDM-based SVM models and by 56.4% compared with the 4-mer MDS. At 95% specificity, the ESM achieved a mean sensitivity of 0.585, exceeding those of the raw EDM (0.418) and MDS (0.195) (Figure 5C). ESM scores were consistently higher in cancer than in non-cancer samples across datasets (Figure S16). Further tumor-burden analysis showed significant differences in ESM scores across clinical stages in two datasets, with average AUCs of 0.770 and 0.787 for stage I and II cancers, respectively (Figure S17A,B and Table S12). Among four datasets with available tumor fraction estimates, three showed significant score differences across low-, medium-, and high-tumor-fraction groups, and the average AUC in the low tumor fraction group was 0.803 (Figure S17C,D and Table S13). Calibration curves showed good agreement between predicted and observed outcomes across most datasets (Figure S18), while decision curve analysis suggested potential clinical utility of the ESM (Figure S19). These findings support the robustness of ESM for cfDNA-based cancer detection across multiple clinical settings.

### 3.5. Frequency-Guided Motif Attribution Reveals Potential Biological Associations

To investigate whether frequency-domain signals can be traced back to biologically interpretable sequence motifs, we performed frequency-guided motif attribution using 4-mer amplitude spectra from the Mathios LUCAS dataset. Significant frequency components were retained and reconstructed into the sequence domain together with phase information, followed by motif-level differential analysis. In total, 46 differential 4-mer motifs (*p* < 0.001) were identified for downstream analysis (Tables S14 and S15).

**Figure 6:**
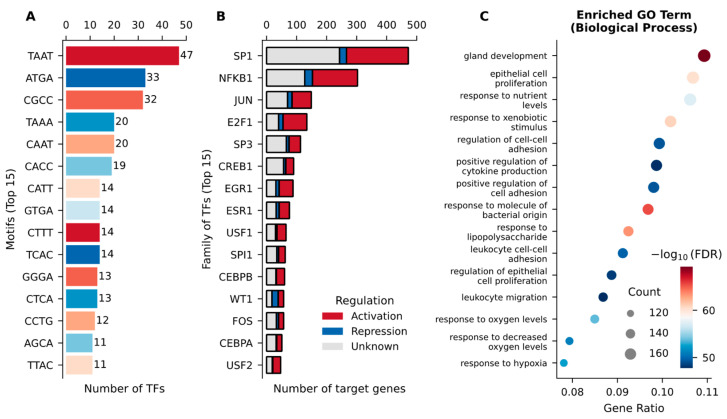
Frequency-guided motif attribution and functional annotation of 4-mer spectral features in the Mathios LUCAS dataset. (**A**) Top differential 4-mer motifs identified after frequency-domain filtering and sequence-domain reconstruction. (**B**) Regulatory landscape of candidate transcription factor (TF) families associated with differential motifs. (**C**) Gene Ontology (GO) biological process enrichment analysis of TF-regulated target genes.

These motifs were subsequently mapped to TFs, resulting in 220 associated regulators. Among them, SP1, NFKB1, JUN, and E2F1 were associated with a relatively large number of target genes in the TF–target regulatory network (Figure 6A,B). Previous studies have implicated SP1 and E2F1 in transcriptional regulation programs associated with lung adenocarcinoma progression, stemness maintenance, and drug resistance [40,41]. NFKB1 and JUN have been linked to inflammatory signaling and transcriptional reprogramming in thoracic malignancies and cancer-related immune responses [42,43]. Together, these findings suggest that the identified motifs may be associated with transcriptional regulators involved in cancer-related biological processes.

GO enrichment analysis of TF-regulated target genes further showed significant enrichment in biological processes related to cell–cell adhesion, epithelial cell proliferation, cytokine production, leukocyte migration, and responses to oxygen levels or hypoxia (Figure 6C and Table S16). These terms broadly overlap with biological processes previously implicated in lung cancer progression [44,45,46], suggesting potential links to tumor invasion, microenvironmental remodeling, and inflammatory or immune-related responses.

Collectively, these findings suggest that DFT-derived spectral features can be traced back to sequence-level motif alterations and may reflect biologically relevant regulatory patterns, providing an interpretable connection between frequency-domain representations and underlying biological signals.

## 4. Discussion

This study presents a frequency-domain framework for cfDNA EDM analysis and shows that transforming raw motif frequency profiles into amplitude spectra improves diagnostic performance across datasets. By providing an alternative representation of EDM features, this framework enhances the separation between cancer and non-cancer samples and improves the robustness of downstream classification.

The observed improvement after frequency-domain transformation may relate to the biological background composition of plasma cfDNA. Since plasma cfDNA is predominantly derived from hematopoietic cells [9,26], EDM profiles may contain substantial background signals that can reduce the detectability of subtle cancer-associated differences. This provides a possible rationale for transforming EDM profiles into alternative feature representations. Consistently, Fourier and wavelet transforms have been applied in cfDNA fragmentomic studies, including tumor fraction prediction from fragment-length distributions [47], cancer detection using wavelet-based representations [48], and classification with Fourier-derived amplitude features from nucleosome profiles [49]. Mathematically, DFT decomposes the original signal into orthogonal frequency components, redistributing correlated variation and enhancing differences between cancer and non-cancer samples. Therefore, the observed benefit should be interpreted as improved feature representation.

Downsampling analysis in the Cristiano dataset indicated that DFT-derived amplitude features were relatively robust to varying sequencing depths (Figure S20). However, the Jiang dataset exhibited several patterns that deviated from the overall trends observed in other datasets, including the reversed MDS pattern, higher 4-mer amplitude spectra in cancer samples, and better performance of some alternative transformation methods and classifiers. Together, these findings suggest that reducing inter-dataset variation while minimizing technical noise remains an important challenge for future work.

We also observed that diagnostic performance did not increase monotonically with motif length. Among the tested features, 5-mer amplitude spectra showed the best overall performance, whereas 6-mer features provided no further improvement. This suggests that increased motif dimensionality does not necessarily enhance discrimination under the current framework, possibly due to increased sparsity or noise in higher-dimensional EDM profiles.

To further evaluate the transformation strategy, we compared DFT with DCT, wavelet transformation, and PCA. DFT showed the most stable overall performance across datasets, while DCT and wavelet performed better in specific datasets, suggesting potential complementarity. Exploratory comparison with PCA showed comparable performance in the validation datasets (Figure S21), indicating that integrating multiple signal representations may be worth exploring in future studies.

This study focused primarily on amplitude spectra for downstream modeling. Although phase is another important component of DFT, additional analyses showed that neither phase-based features nor their combination with amplitude features consistently improved diagnostic performance (Figures S22 and S23). Under the current framework, the main discriminative information appears to be captured by amplitude features, while the contribution of phase information remains limited.

The influence of motif ordering on frequency-domain analysis warrants consideration. There is currently no clear evidence that motif ordering itself carries direct biological meaning. Our permutation analysis suggests that the default lexicographic ordering provides a stable basis for DFT analysis, though alternative motif arrangements may generate different spectral patterns. The relationship between motif ordering, spectral decomposition, and biological interpretation remains to be further explored.

Several limitations should also be acknowledged. Although the proposed framework improved performance relative to the raw EDM and MDS, performance in the Mathios independent dataset remained modest, suggesting persistent inter-dataset variation. In addition, the biological interpretation of frequency-domain signals remains preliminary, and some TF and functional associations may be weak or non-specific. A further limitation is that cancer-specific analyses in the Cristiano dataset were limited, as the study focused on pan-cancer analyses. Nevertheless, additional analysis in the BRCA subset showed that the ESM remained superior to the raw EDM-based model (Figure S24), suggesting its potential utility in single-cancer analysis. Finally, this study focused specifically on EDM-based analysis and did not systematically compare amplitude features with other fragmentomic modalities. Future studies may explore multimodal frameworks integrating DFT-derived amplitude features with other cfDNA characteristics.

## 5. Conclusions

In this study, we systematically evaluated the limitations of raw EDM features across six datasets and introduced a frequency-domain analytical framework for EDM signal characterization. Compared with conventional EDM-based approaches, DFT-derived amplitude features showed improved diagnostic performance and cross-dataset stability. By integrating multi-scale spectral features through the ESM, diagnostic performance was further improved across datasets. In addition, frequency-guided motif attribution linked spectral features back to sequence-level motif patterns and may provide potential biological context for the observed signal differences.

Collectively, these findings highlight a new computational strategy that leverages frequency-domain transformation to enhance EDM-based cancer detection across diverse cfDNA datasets.

## Figures and Tables

**Table 1:** Data information for all datasets.

Dataset	Total	Controls	Cancer Types	Clinical Stage	Evaluation
Mathios et al. LUCAS	287	Healthy (91), Benign (67)	Lung (129)	I (15), II (7), III (35), IV (72)	Cross-validation
Mathios et al. independent	431	Healthy (385)	Lung (46)	I (28), II (12), III (5), IV (1)	Independent validation
Yu et al. study	249	Healthy (130), CNAG (8), CAG (1)	Gastric (110)	I (85), II (25)	Cross-validation
Yu et al. validation	167	Healthy (80), CNAG (10), CAG (4)	Gastric (73)	I (56), II (17)	Independent validation
Cristiano et al.	423	Healthy (215)	BRCA (54), PAAD (34), OV (28), CRC (27), STAD (27), CHOL (26), NSCLC (12)	I (41), II (109), III (33), IV (22), X (3)	Cross-validation
Jiang et al.	225	Healthy (32), HBV (67), Cirrhosis (36)	HCC (90)	NA	Cross-validation
Total	1782	Non-Cancer (1126)	Cancer (656)	I (225), II (170), III (73), IV (95), X (3)	Cross-validation

Numbers in parentheses indicate the number of samples in each group. X indicates unknown clinical stage. NA indicates not available. Abbreviations: CNAG, chronic non-atrophic gastritis; CAG, chronic atrophic gastritis; HBV, hepatitis B virus; BRCA, breast cancer; PAAD, pancreatic adenocarcinoma; OV, ovarian cancer; CRC, colorectal cancer; STAD, stomach adenocarcinoma; CHOL, cholangiocarcinoma; NSCLC, non-small cell lung cancer; HCC, hepatocellular carcinoma.

## Data Availability

The datasets analyzed in this study were obtained from publicly available datasets from previously published studies, including the Mathios et al. lung cancer study (LUCAS and independent datasets; EGA accession: EGAS00001005340), the Yu et al. gastric cancer study (study and validation datasets; NGDC accession: PRJCA020703), and the Cristiano et al. pan-cancer dataset together with the Jiang et al. hepatocellular carcinoma dataset retrieved from FinaleDB, as cited in the main text. No new data were generated in this study. All processed data used in this study, including EDM feature matrices, sample annotation tables, and tumor fraction estimates, are publicly available in the project GitHub repository. The source code for data processing, signal transformation, model construction, statistical analysis, and figure generation is also freely available at: https://github.com/Upupdownn/DFT_code (version 1.0; accessed on 4 June 2026).

## Supplementary Materials

The following supporting information can be downloaded at: https://www.mdpi.com/article/10.3390/genes17060661/s1.

## Abbreviations

The following abbreviations are used in this manuscript:

AUC	Area under the Receiver Operating Characteristic Curve
BH	Benjamini–Hochberg
cfDNA	Cell-free DNA
CI	Confidence Interval
DCT	Discrete Cosine Transform
DFT	Discrete Fourier Transform
EDM	End-Motif
ESM	Ensemble Spectral Model
FDR	False Discovery Rate
GBDT	Gradient Boosting Decision Trees
GO	Gene Ontology
LR	Logistic Regression
MDS	Motif Diversity Score
RF	Random Forest
SVM	Support Vector Machine
TF	Transcription Factor
WGS	Whole-Genome Sequencing
