# Supplementary material for: Frequency-Domain Transformation of cfDNA End-Motif Profiles Enhances Robust Cancer Detection
Source: Genes (Basel). 2026 Jun 5;17(6):661. doi: 10.3390/genes17060661 (PMC13298836; doi:10.3390/genes17060661)
Supplement: Supplementary file 1 [file genes-17-00661-s001.zip › Supplementary_Methods_and_Figures.pdf]

Supplementary Information

Frequency-Domain Transformation of cfDNA End-Motif Profiles Enhances Robust Cancer Detection

Xinwei Sheng, Xinming Du, Qianqian Shi and Xionghui Zhou *

College of Informatics, Huazhong Agricultural University, Wuhan 430070, Hubei, China
* Correspondence: zhouxionghui@mail.hzau.edu.cn; Tel.: +86-13349913458

Supplementary Methods

S1. Comparative Signal Transformation Methods

To benchmark the Discrete Fourier Transform (DFT)-based transformation strategy, Discrete Cosine Transform (DCT) and wavelet transformation were additionally applied to raw end-motif (EDM) frequency profiles.

S1.1. Discrete Cosine Transform (DCT)

DCT was applied directly to the raw EDM frequency vector of each sample using the dct function from the SciPy library (scipy.fft.dct, SciPy v1.14.1). Transformation was performed using the default type-II DCT with orthogonal normalization (norm='ortho'). The resulting DCT coefficients were used directly as input features for downstream classification analysis.

S1.2. Wavelet Transform

Wavelet transformation was performed directly on raw EDM frequency vectors using the PyWavelets library (pywt, v1.8.0). Signal decomposition was conducted using the Daubechies-4 (db4) wavelet. The decomposition level was set to None, allowing the maximum valid level to be determined automatically according to signal length. Boundary extension was performed using mode='symmetric'. Approximation and detail coefficients from all decomposition levels were concatenated and used as features for downstream classification analysis.

S2. Model Implementation, Permutation Validation, and Confidence Interval Estimation

S2.1. Model Implementation and Hyperparameter Settings

All machine learning models were implemented using the scikit-learn library (v1.7.1) in Python (v3.10). Unless otherwise specified, default hyperparameters were used.

Support Vector Machine (SVM) was implemented using sklearn.svm.SVC with a linear kernel

and a penalty parameter of C = 1.0. Logistic Regression (LR) was implemented using sklearn.linear_model.LogisticRegression with default settings, including L2 regularization, C = 1.0, and the “lbfgs” solver. Random Forest (RF) was implemented using sklearn.ensemble.RandomForestClassifier with default parameters, including n_estimators = 100, criterion = 'gini', and max_features = 'sqrt'. Gradient Boosting Decision Trees (GBDT) was implemented using sklearn.ensemble.GradientBoostingClassifier with default parameters, including n_estimators = 100, learning_rate = 0.1, and subsample = 1.0. The default maximum depth of the individual regression trees was 3.

S2.2. Permutation-Based Validation of Motif Ordering

Permutation-based validation was performed to assess the effect of motif ordering on DFT-derived features. For each permutation, the column order of the EDM feature matrix was randomly shuffled prior to DFT transformation, while maintaining the same permutation across all samples within the dataset to preserve sample-to-sample correspondence. Since rows represented samples and columns represented motif features, this procedure altered motif ordering while preserving the original feature values of each sample.

Column permutation was implemented using the permutation function from NumPy’s random number generator (numpy.random.default_rng). A total of 1,000 permutations were performed, with the random seed set to the permutation index (0–999) for reproducibility. For datasets with matched independent validation sets, the same column permutation order generated from the corresponding training dataset was applied to the validation dataset. Following permutation, DFT transformation, feature extraction, model training, and evaluation were performed using the same workflow as described in the main Methods section. Area Under the Curve (AUC)

values from all permutations were collected for downstream analysis.

S2.3. Confidence Interval Estimation by Bootstrap Resampling

Confidence intervals (95% CI) for AUC and sensitivity were estimated using bootstrap resampling with 1,000 iterations. For the primary diagnostic analyses, standard bootstrap resampling with replacement was performed at the sample level. For each bootstrap iteration, AUC was calculated based on the resampled predictions. Sensitivity at fixed specificity was calculated by linear interpolation using the numpy.interp function.

The 95% confidence intervals were determined using the 2.5th and 97.5th percentiles of the bootstrap distribution with the numpy.percentile function.

For subgroup analyses involving cancer stage and tumor fraction, stratified bootstrap resampling was additionally applied to preserve the proportion of samples in each subgroup due to limited sample size. Unless otherwise specified, bootstrap resampling was performed with a fixed random seed of 42.

Supplementary Figures

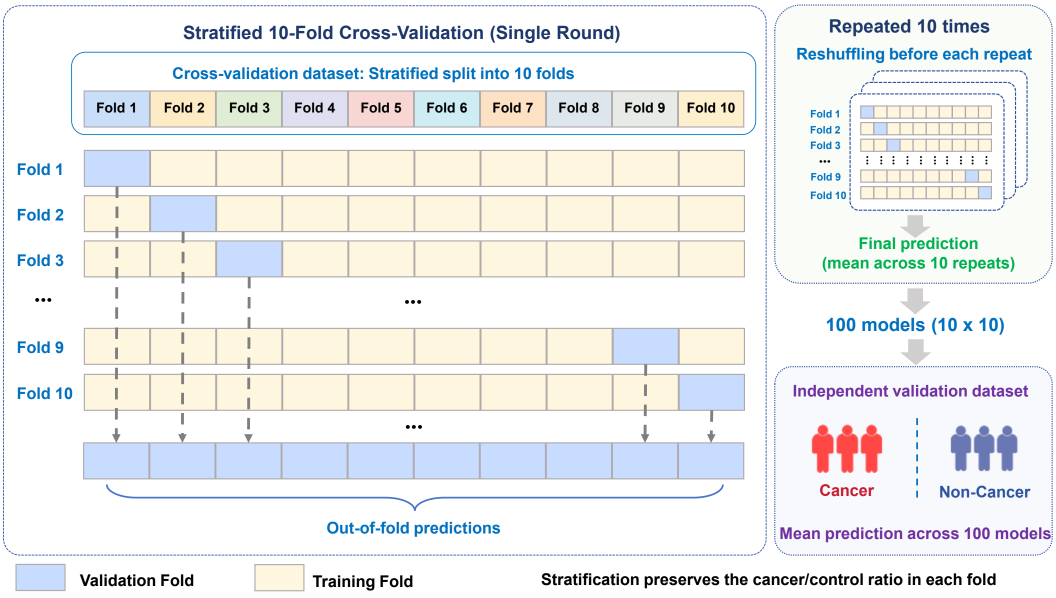

Figure S1. Schematic illustration of the repeated stratified 10-fold cross-validation workflow.

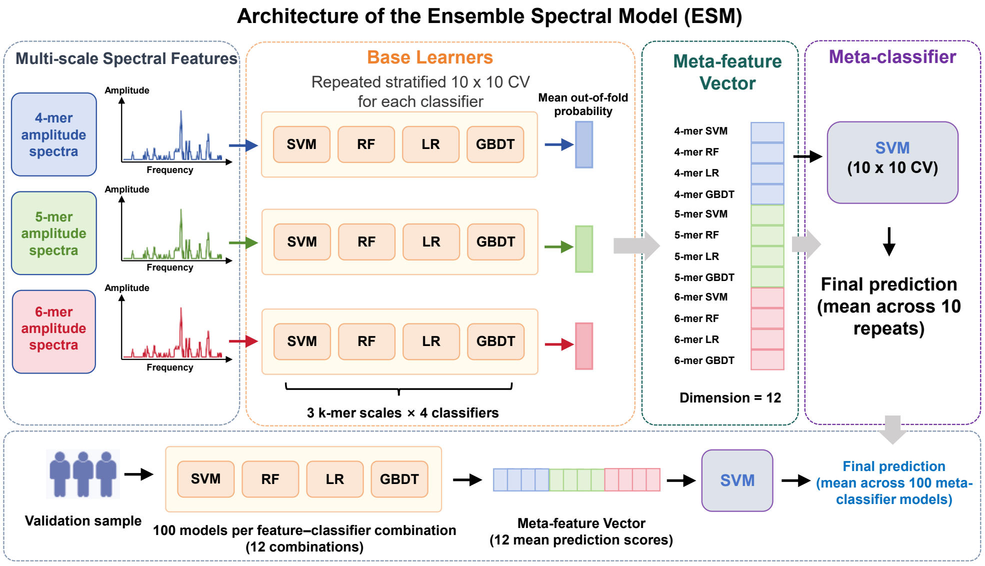

Figure S2. Schematic overview of the Ensemble Spectral Model (ESM) architecture and prediction workflow.

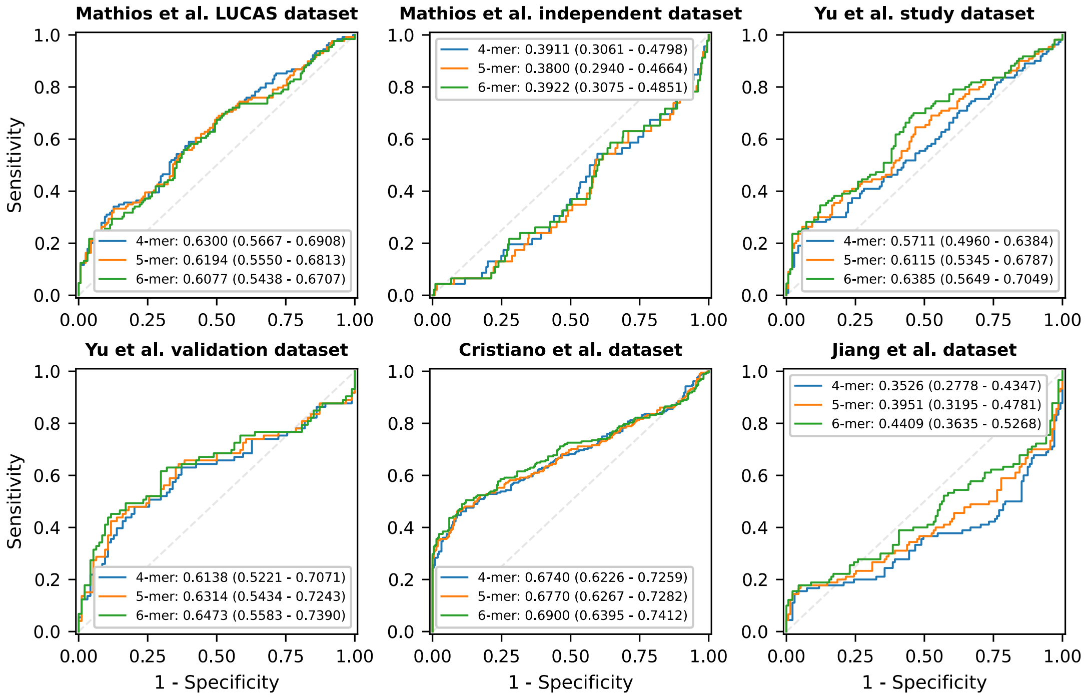

Figure S3. Cross-dataset ROC analysis of MDS derived from 4–6-mer EDM profiles.

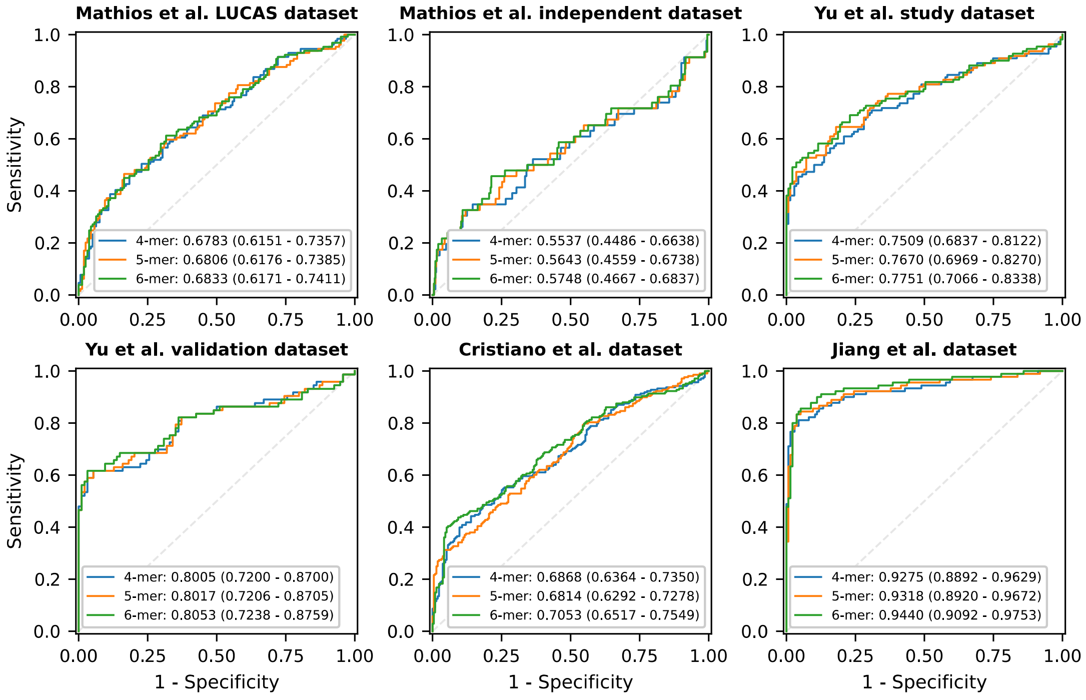

Figure S4. Cross-dataset ROC analysis of SVM models based on 4–6-mer EDM profiles.

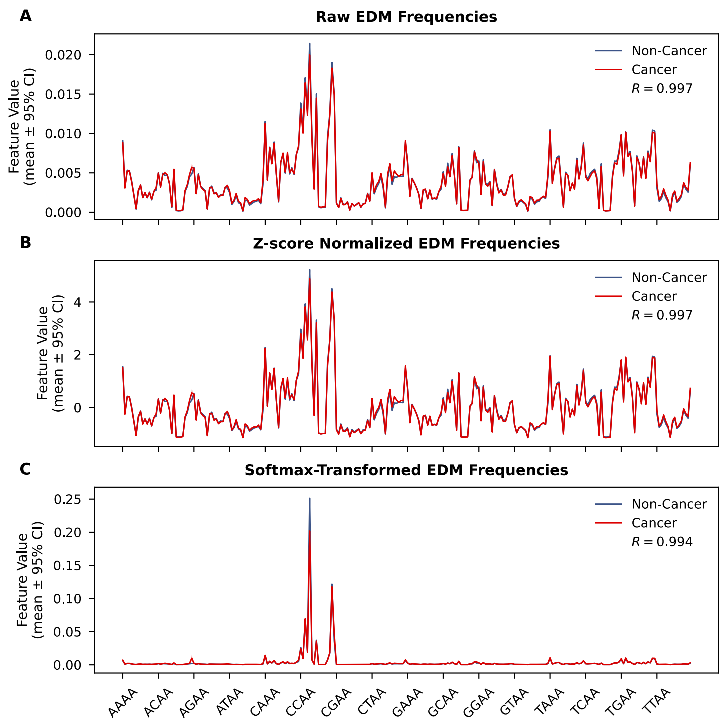

Figure S5. Preprocessing workflow of EDM features. Representative examples are shown using 4-mer EDM features from the Cristiano dataset.

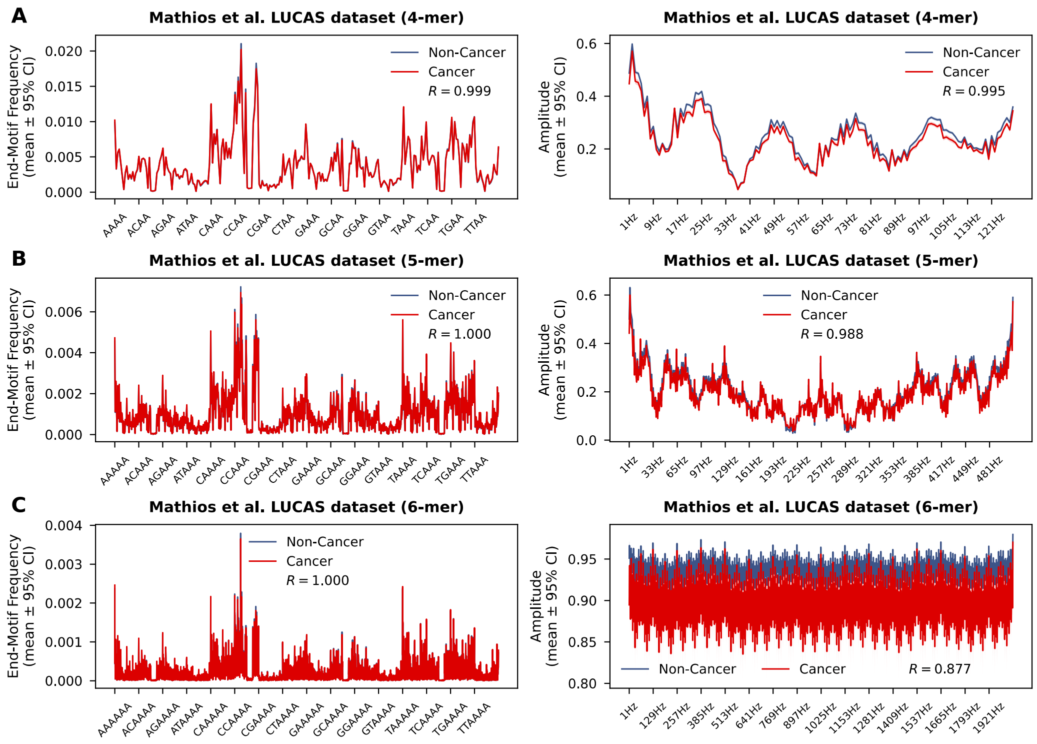

Figure S6. Frequency-domain transformation of EDM profiles in the Mathios et al. LUCAS dataset. (A–C) Raw EDM frequency profiles and corresponding amplitude spectra for 4-mer (A), 5-mer (B), and 6-mer (C) motifs.

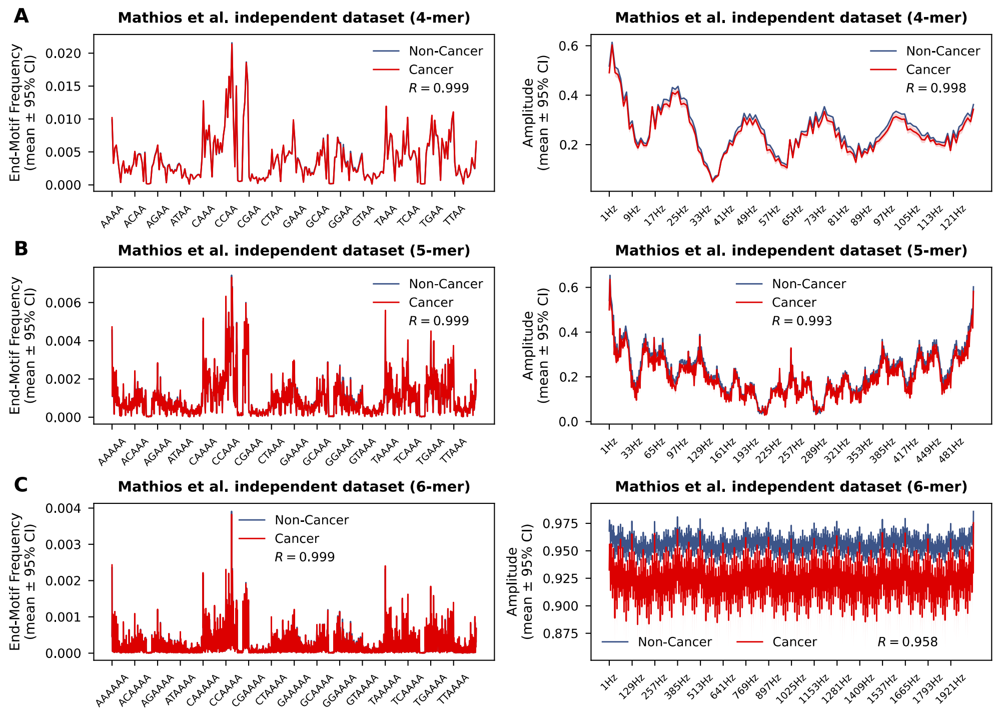

Figure S7. Frequency-domain transformation of EDM profiles in the Mathios et al. independent dataset. (A–C) Raw EDM frequency profiles and corresponding amplitude spectra for 4-mer (A), 5-mer (B), and 6-mer (C) motifs.

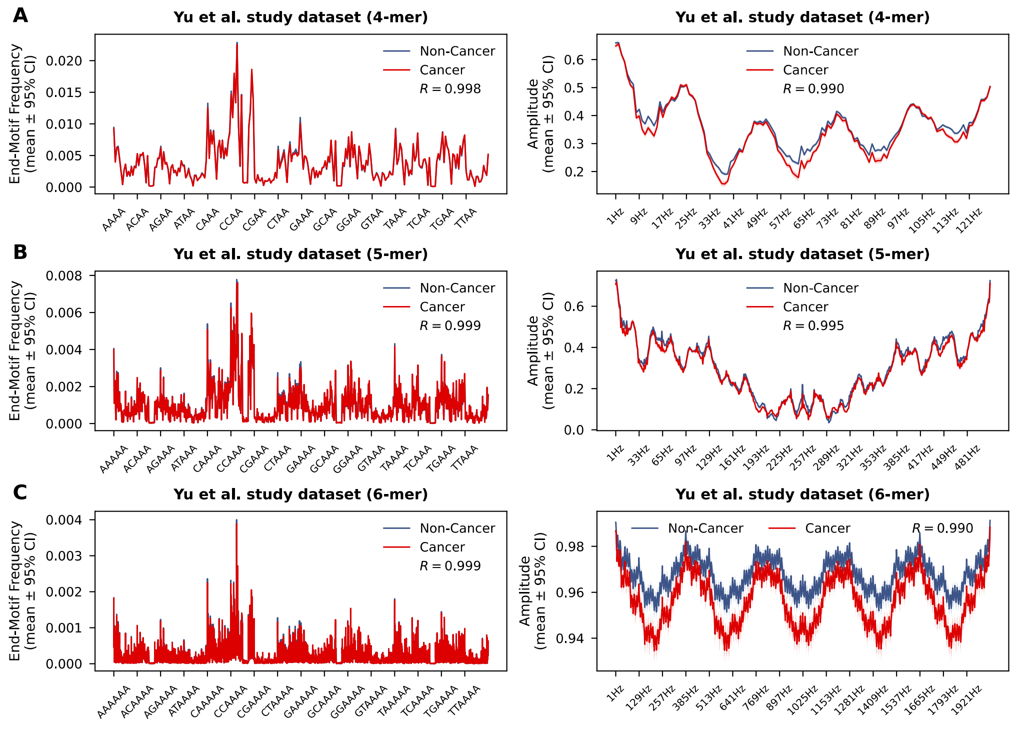

Figure S8. Frequency-domain transformation of EDM profiles in the Yu et al. study dataset. (A–C) Raw EDM frequency profiles and corresponding amplitude spectra for 4-mer (A), 5-mer (B), and 6-mer (C) motifs.

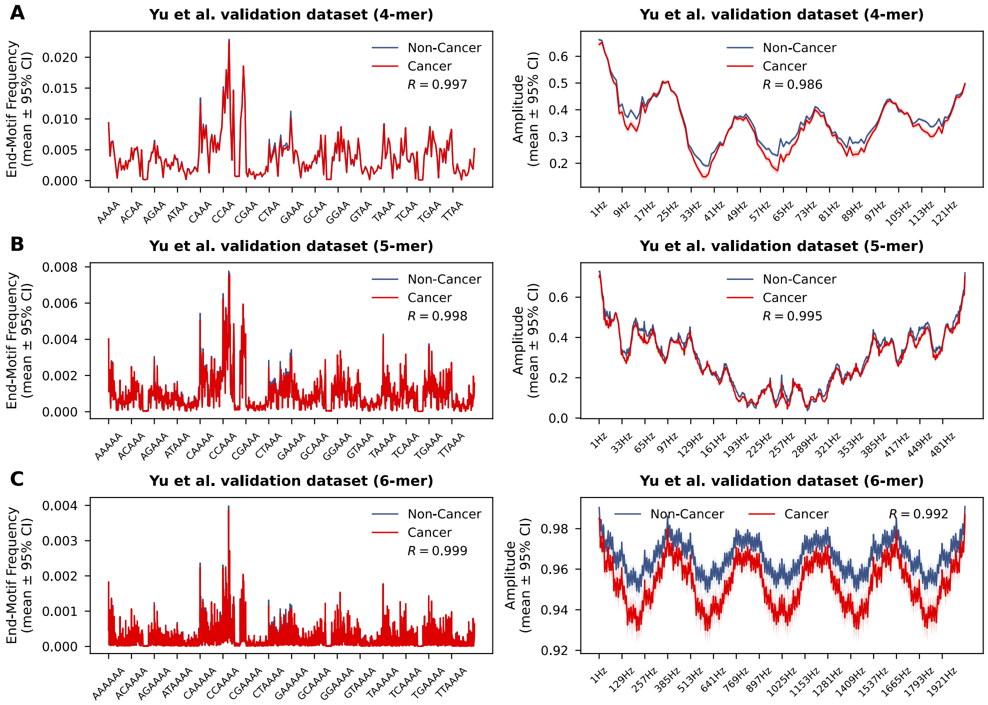

Figure S9. Frequency-domain transformation of EDM profiles in the Yu et al. validation dataset. (A–C) Raw EDM frequency profiles and corresponding amplitude spectra for 4-mer (A), 5-mer (B), and 6-mer (C) motifs.

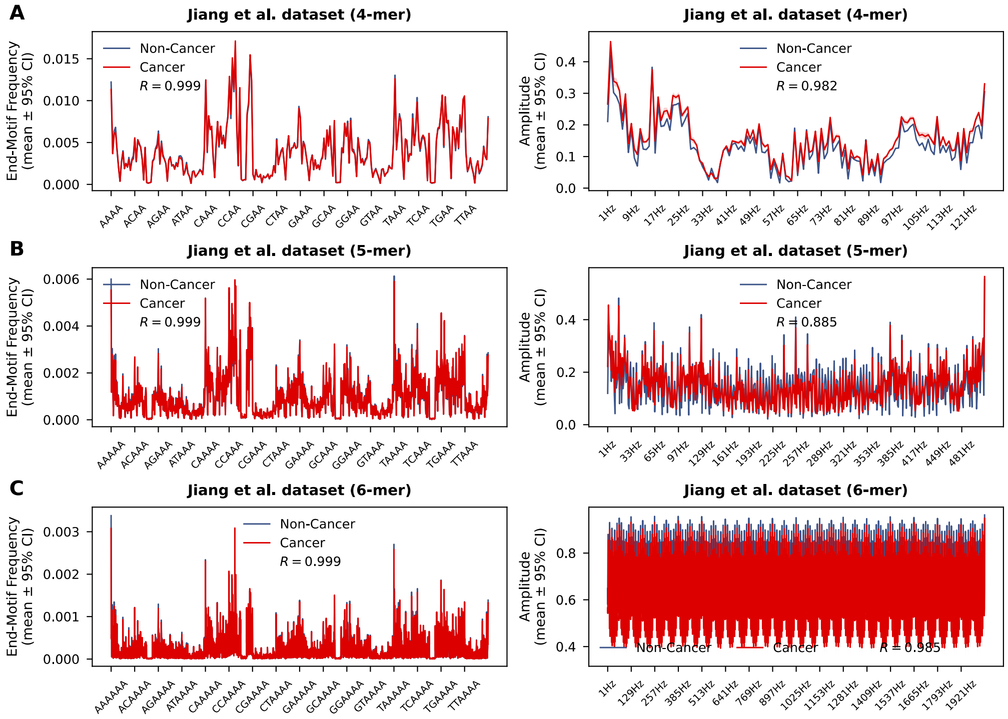

Figure S10. Frequency-domain transformation of EDM profiles in the Jiang et al. dataset. (A–C) Raw EDM frequency profiles and corresponding amplitude spectra for 4-mer (A), 5-mer (B), and 6-mer (C) motifs.

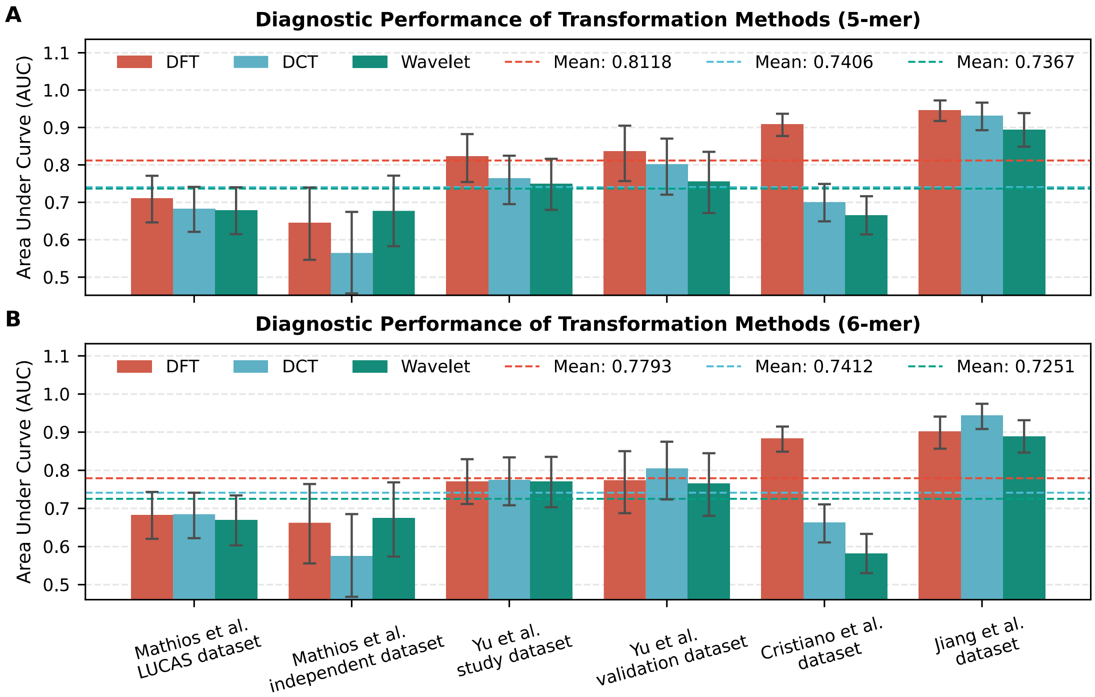

Figure S11. Comparison of signal transformation methods based on 5-mer (A) and 6-mer (B) EDM features using SVM across six datasets.

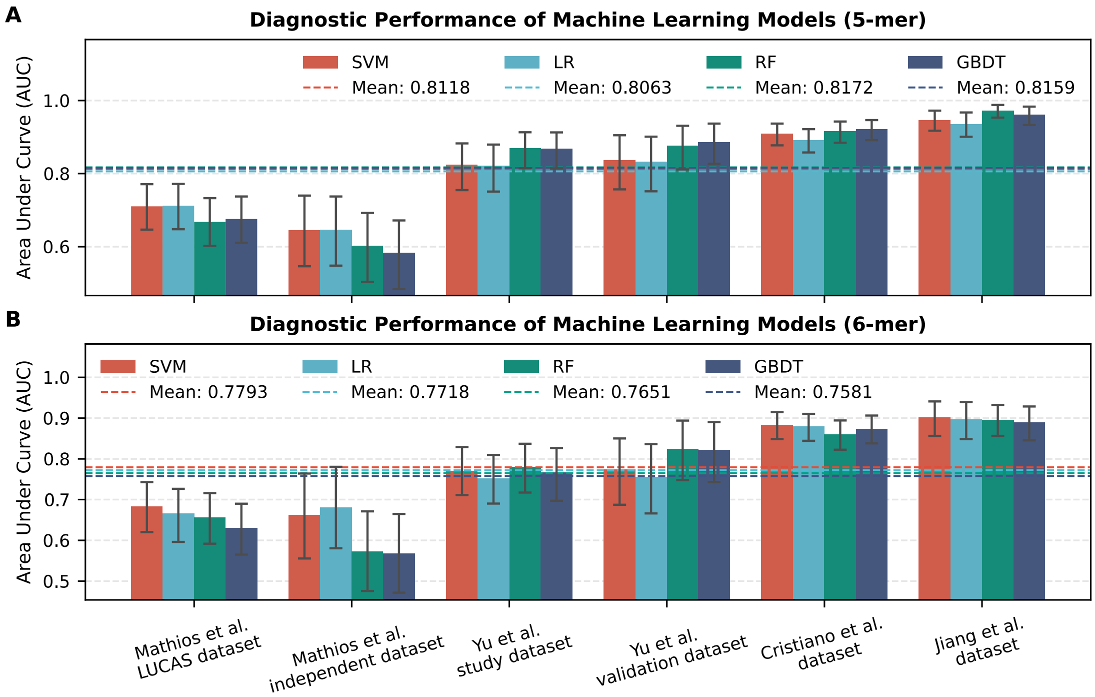

Figure S12. Comparison of machine learning models based on 5-mer (A) and 6-mer (B) amplitude spectrum features across six datasets.

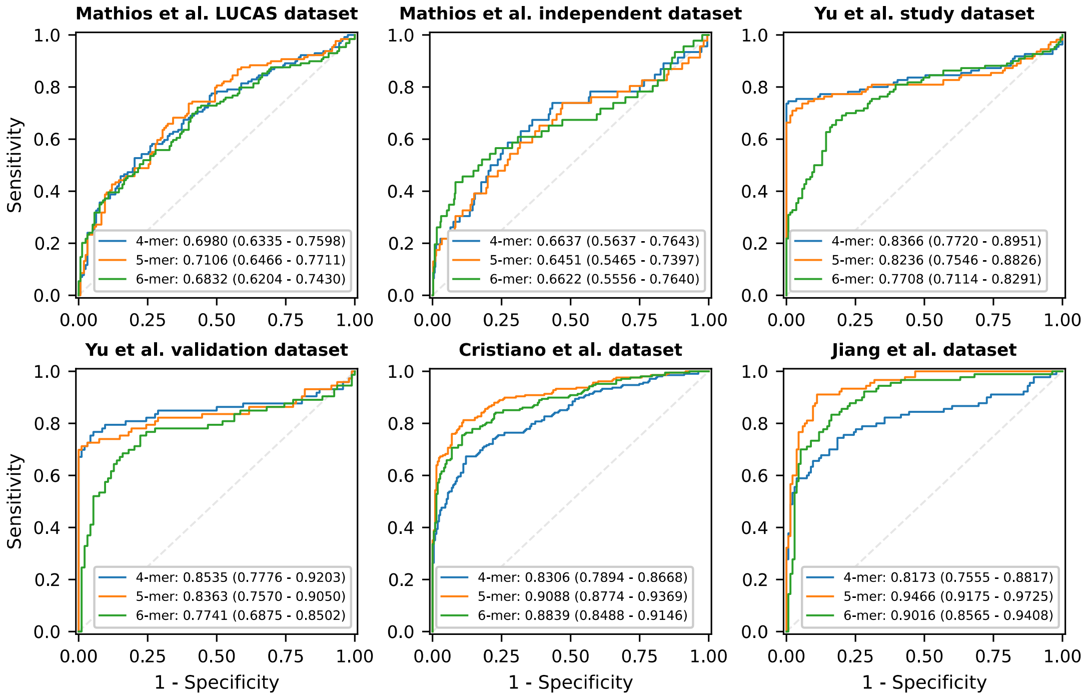

Figure S13. Cross-dataset ROC analysis of SVM models based on amplitude spectra of 4–6-mer EDM profiles.

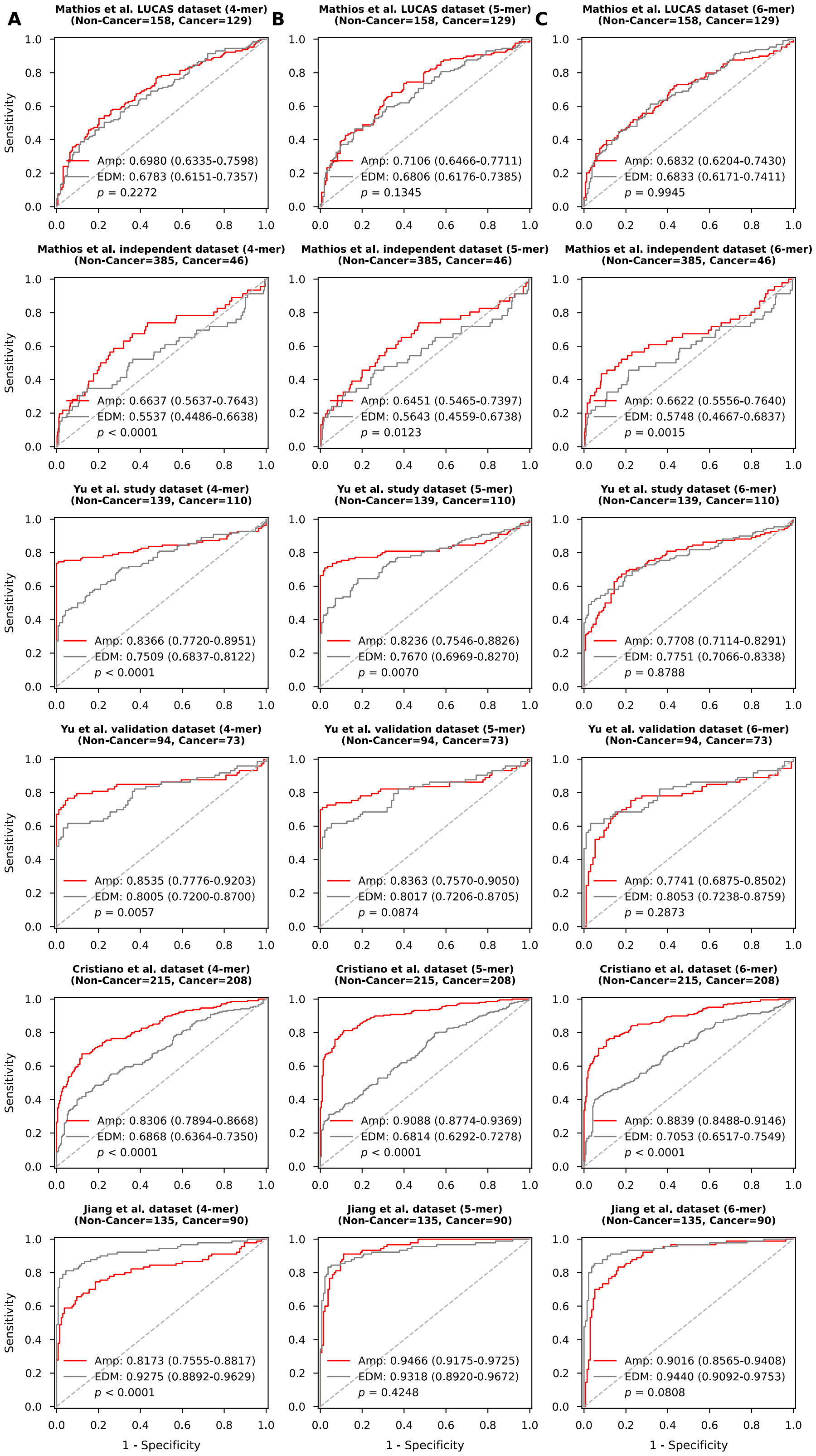

Figure S14. Cross-dataset ROC analysis comparing SVM models based on raw EDM profiles and amplitude spectra of 4–6-mer EDM features. DeLong test *p* values are shown in each ROC plot to assess differences in diagnostic performance between the two models.

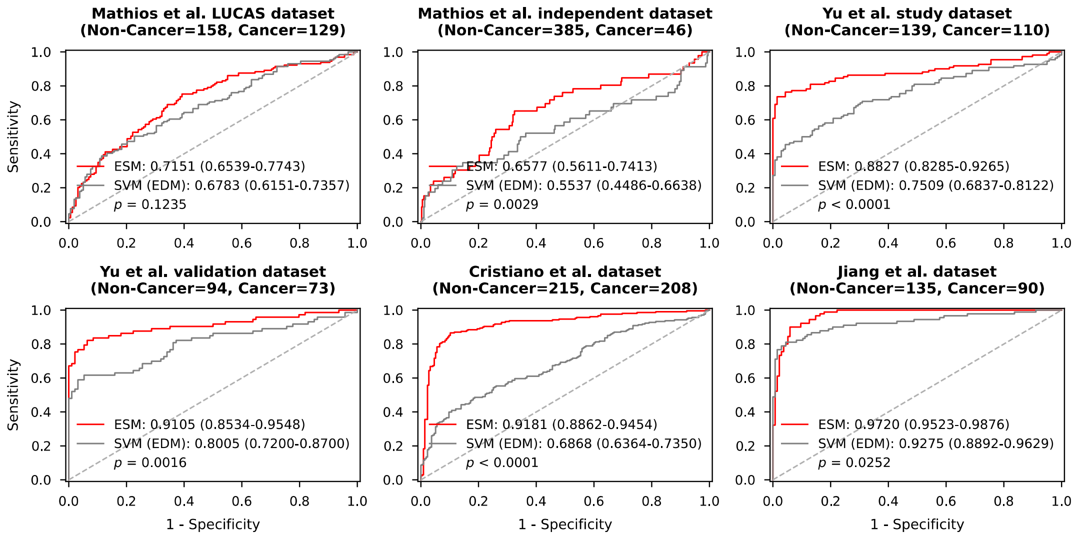

Figure S15. Cross-dataset ROC analysis comparing the ESM and the SVM model based on 4-mer raw EDM profiles.

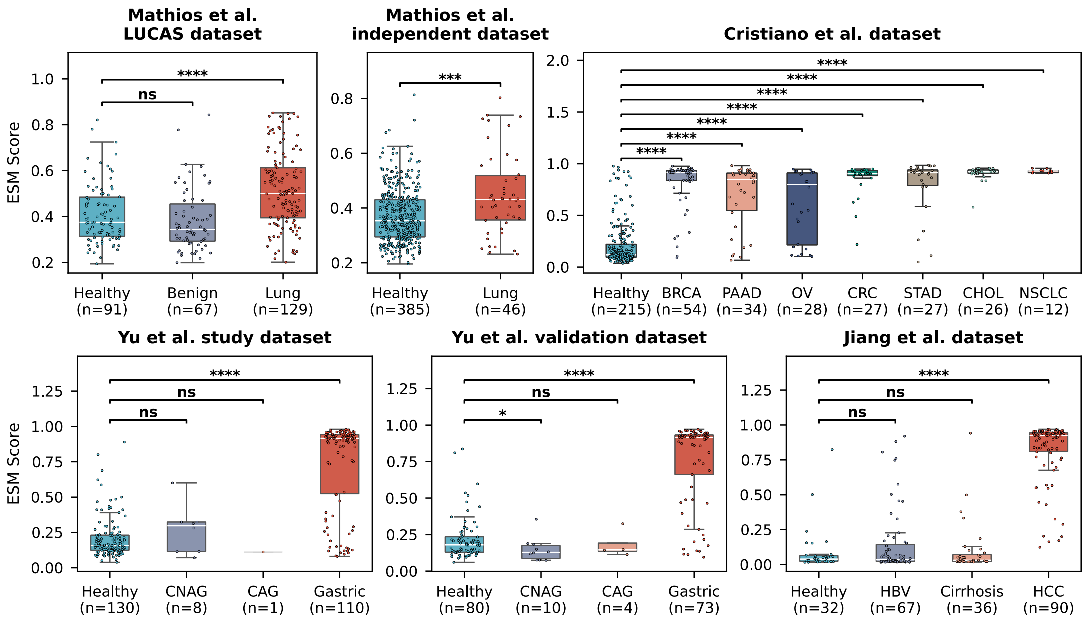

Figure S16. Distribution of ESM scores across different datasets and clinical groups. Statistical significance between groups was assessed using the Wilcoxon rank-sum test, whereas comparisons among three or more groups were performed using the Kruskal–Wallis test. Significance levels are denoted as ns (not significant), * $P < 0.05$, ** $P < 0.01$, *** $P < 0.001$, and **** $P < 0.0001$.

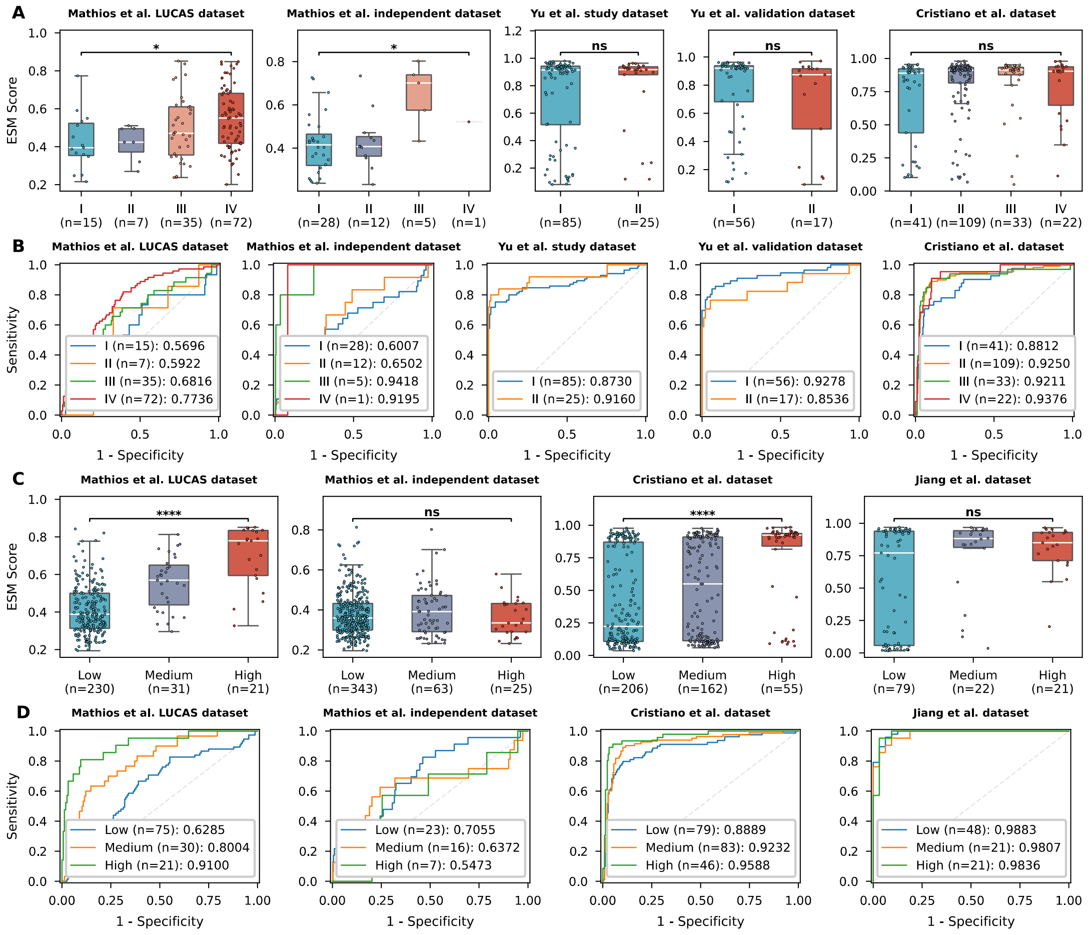

Figure S17. Evaluation of ESM performance across clinical stages and tumor fractions. (A) Distribution of ESM scores stratified by clinical stage. (B) ROC analysis of the ESM across different clinical stages. (C) Distribution of ESM scores stratified by tumor fraction groups. (D) ROC analysis of ESM across different tumor fraction groups. Tumor fraction groups were defined as low (≤2%), medium (>2% and ≤15%), and high (>15%).

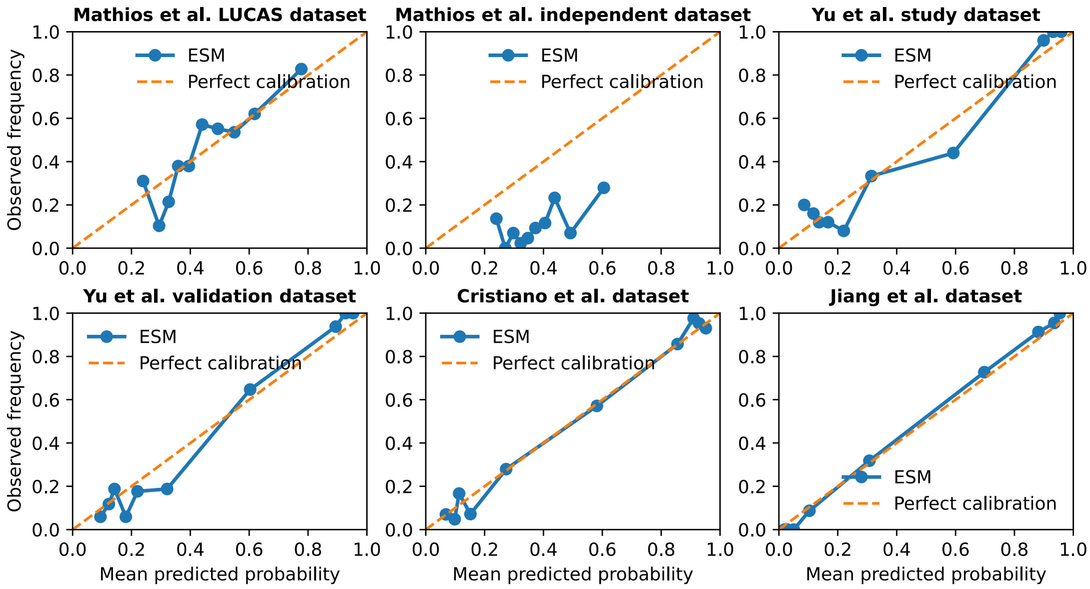

Figure S18. Calibration curves of the ESM across six datasets. The dashed diagonal line indicates perfect calibration.

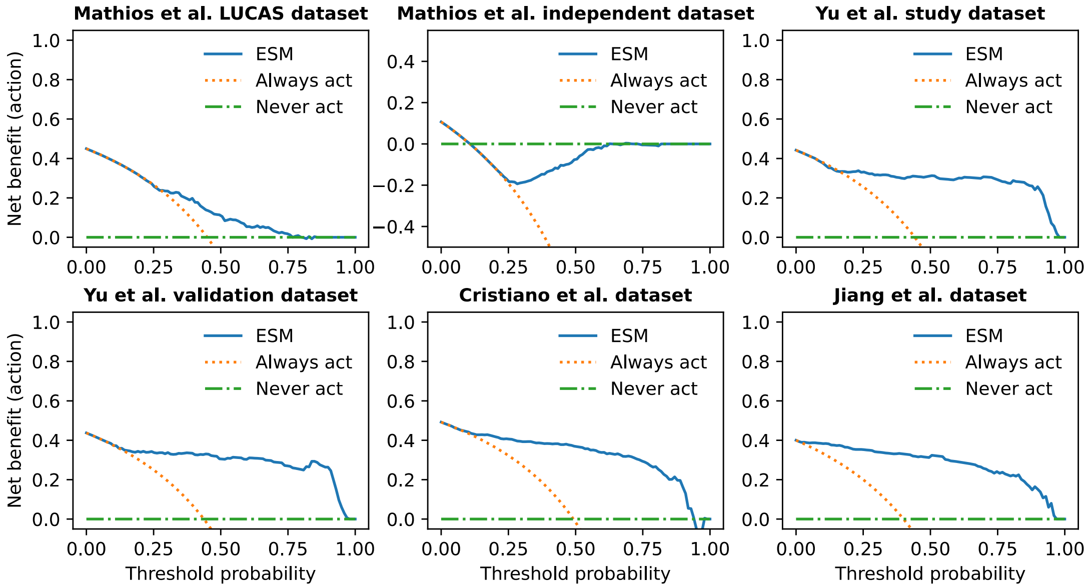

Figure S19. Decision curve analysis of the ESM across six datasets. The dotted orange line represents the strategy of treating all samples as cancer ("Always act"), and the green dash-dot line represents the strategy of treating no samples as cancer ("Never act").

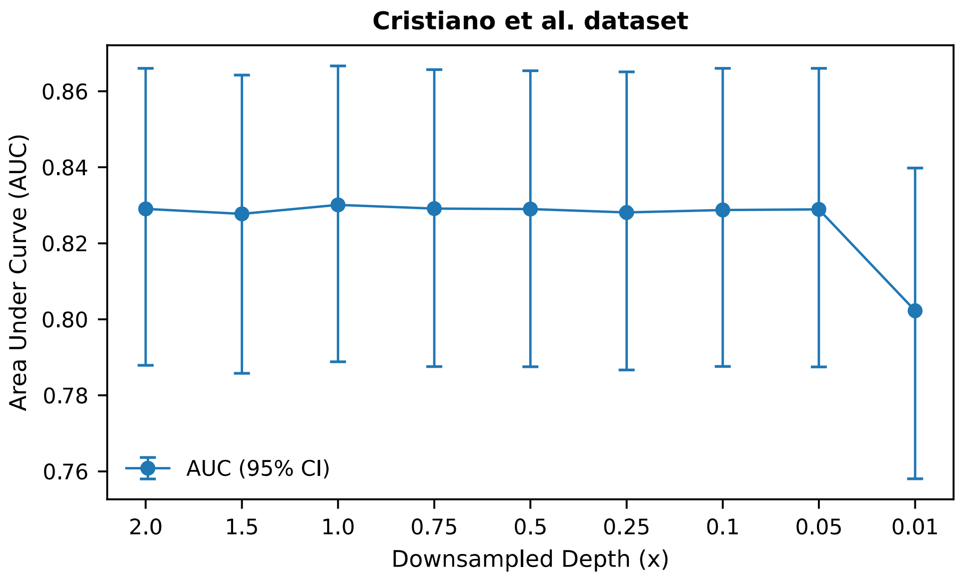

Figure S20. Performance of the downsampling analysis across different sequencing depths in the Cristiano et al. dataset. The AUC values of the SVM classifier based on 4-mer DFT amplitude spectrum features are shown with 95% confidence intervals.

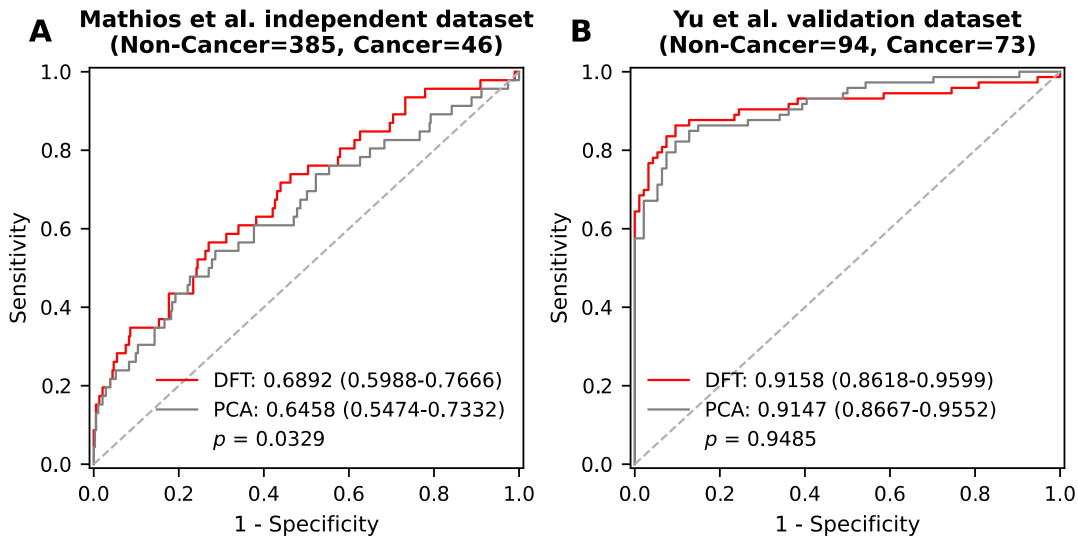

Figure S21. ROC comparison of DFT- and PCA-based SVM models in independent validation datasets. ROC curves comparing SVM models constructed using DFT-derived amplitude spectrum features and PCA-transformed EDM features in the Mathios independent dataset (A) and Yu validation dataset (B). PCA was fitted on the corresponding training datasets (Mathios LUCAS and Yu study datasets) with 95% explained variance retained, and the transformed validation datasets were subsequently evaluated using the trained SVM models. DFT-based models were constructed using amplitude spectrum features following the same training and validation workflow.

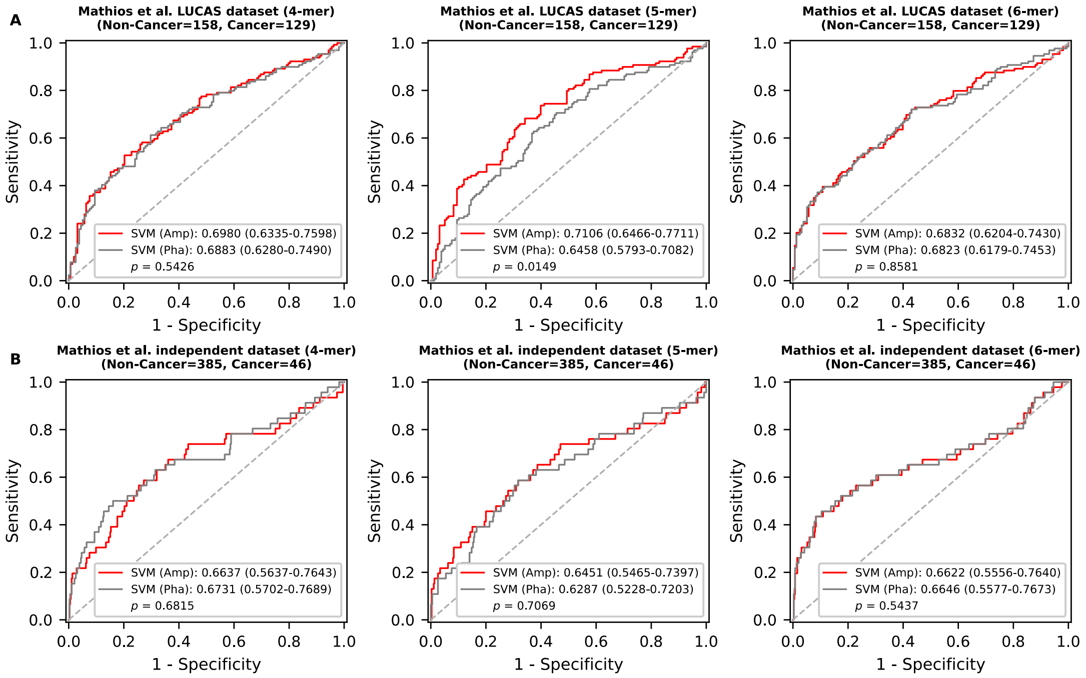

Figure S22. ROC comparison of SVM models based on amplitude (Amp) and phase (Pha) spectrum features in the Mathios LUCAS dataset (A) and Mathios independent dataset (B). For phase-spectrum analysis, the same non-redundant frequency components as used for amplitude features were retained, and phase values were transformed by sine and cosine encoding before concatenation for model construction.

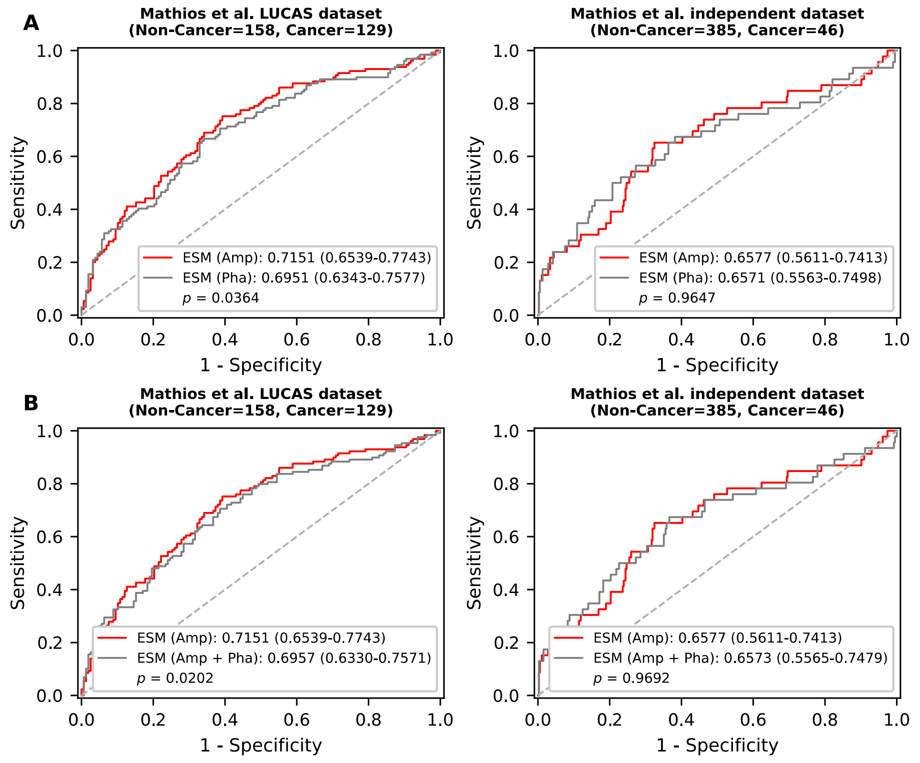

Figure S23. ROC comparison of ESMs based on different spectral feature representations in the Mathios et al. datasets. (A) Comparison between the amplitude-based ESM (Amp) and the phase-based ESM (Pha). (B) Comparison between the amplitude-based ESM (Amp) and the ESM using combined amplitude and phase features (Amp + Pha). For the Amp + Pha model, the model architecture and training workflow were identical to those used for the default Amp-based ESM, with the number of input feature sets increasing from three to six, resulting in a 24-dimensional meta-feature vector.

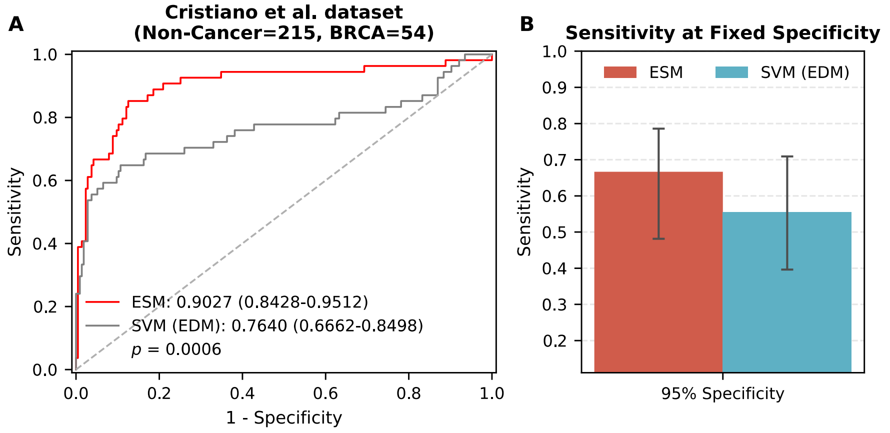

Figure S24. Cancer-type-specific performance analysis for breast cancer (BRCA) samples in the Cristiano et al. dataset. (A) ROC curve comparison between the ESM model and the SVM model based on raw 4-mer EDM features. (B) Sensitivity comparison at 95% specificity.
